# Cesarean Delivery in a Patient With Body Mass Index Over 100: Continuous Spinal Anesthesia in Two Consecutive Deliveries

**DOI:** 10.7759/cureus.15643

**Published:** 2021-06-14

**Authors:** Joseph L Reno, Meghan I Cook, Michael Kushelev, Blair H Hayes, John Coffman

**Affiliations:** 1 Anesthesiology and Pain Medicine, University of Washington, Seattle, USA; 2 Anesthesiology, The Ohio State University, Columbus, USA; 3 Anesthesiology, The Ohio State University Wexner Medical Center, Columbus, USA

**Keywords:** obesity, cesarean, intrathecal catheter, postdural puncture headache, team coordination, continuous spinal anesthesia

## Abstract

Anesthetic implications for morbidly obese parturients have been well described; however, the literature has not yet clarified whether there are additional or unique concerns if the body mass index (BMI) rises farther above the so-called super morbid obesity level: BMI >50 kg/m^2^. There have only been a few case reports focusing on patients with BMI close to or above 100. Parturients with BMI significantly greater than 50 are uncommon, but they represent an increasing proportion among the morbidly obese. In this report, we present the use of continuous spinal anesthesia in consecutive cesarean deliveries for a patient with a BMI of 102 at her first delivery and 116 at her second. For both deliveries, an intrathecal catheter dosing incrementally provided effective anesthesia with a cumulative dose of hyperbaric bupivacaine 12 mg, fentanyl 15 mcg, and morphine 100 mcg given in 0.25-ml increments over 12 minutes, with 0.25-ml sterile saline flushes between doses. While dosing the catheter, the patient was gradually lowered to a 30° semi-recumbent position for surgery. This strategy minimized the risk of high spinal block or respiratory distress. She did not develop any postdural puncture headache (PDPH). This case report offers an extreme example and provides estimates towards adjusting staffing, equipment, location, timing, positioning, anesthetic technique, and dosing for cesarean deliveries in patients with very high BMI levels.

## Introduction

The global prevalence of obesity is steadily on the rise, with a recent report stating that nearly one-third of the world’s population is overweight [body mass index (BMI) >25 kg/m^2^] or obese (BMI >30) [1]. In the United States, 37% of women aged 20-39 years are obese (BMI >30) and 10% are morbidly obese (BMI >40) [2]. Studies rarely stratify BMI of above 50 kg/m^2^, and BMIs significantly higher than that receive even less attention, although these individuals represent an increasing proportion among obese patients [3].

Morbid obesity in pregnant women adds several challenges to their obstetric and anesthetic management [4]. Compared to non-obese parturients, morbidly obese patients are more likely to face cesarean delivery, longer operative times, invasive monitoring, prolonged and difficult neuraxial block placement, higher block failure rates, and if general anesthesia is required, difficult or failed intubation [4-7]. Additionally, morbidly obese parturients usually have major comorbidities, including restricted ventilation, obstructive sleep apnea (OSA), diabetes mellitus, preeclampsia, hypertension, or thromboembolic disorders [4,5].

Studies on the obstetric anesthetic management of patients with very high BMI are scarce in the literature. There is no consensus as to whether risks rise continuously, plateau, or show an inflection point as BMI rises above 40. Our literature review has revealed only a single case report discussing general anesthesia during a repeat cesarean delivery in a woman with BMI >100, and three case series discussing five cesarean patients with BMI ranging from 70 to 90 [8-11]. In this report, we describe the anesthetic management of a patient with a BMI >100 during consecutive cesarean deliveries, focusing on considerations unique to cases with this level of obesity.

Informed, written consent from the patient was obtained for the publication of the details of this case and is kept on file at our institution.

## Case presentation

First delivery

A 20-year-old, G1P0 woman at 39 weeks and two days of gestation was admitted prior to her scheduled primary cesarean delivery for breech presentation. Two weeks before her admission, she had consulted with an obstetric anesthesiologist. Her medical history was notable for morbid obesity (BMI: 102, height: 165 cm, weight: 279 kg), chronic hypertension, asthma, OSA, and a pulmonary embolism at 15 weeks of gestation. She was adherent to a daily, therapeutic dose of enoxaparin, and on admission, she transitioned to a heparin infusion, which was stopped 10 hours before the surgery. She reported longstanding dyspnea while supine, both before and throughout pregnancy. She could breathe comfortably in a 30° semi-upright position. Preoperatively, she underwent ultrasound-guided placement of a radial arterial line and two 18-gauge (G) peripheral IV lines.

In the obstetric operating room (OR), she was placed in a seated position on the operating table for placement of the neuraxial catheter. Electrocardiogram, pulse oximetry (SpO_2_), and blood pressure monitoring were initiated. A 4-inch-wide medical tape used horizontally held her skin folds pulled laterally, and her spinous processes were palpated. An intrathecal catheter was planned to allow incremental dosing up to T4-T6 sensory level and minimize the risk of high neuroblockade or respiratory distress. The catheter also provided an option for redosing, in case of prolonged surgery. The patient strongly preferred neuraxial anesthesia, though the possibility of general endotracheal anesthesia was also discussed with her in the event that neuraxial block proved impossible or impractical. Her airway exam was reassuring, revealing Mallampati class I, full cervical range of motion, and normal oral opening and thyromental distance. Positioning for and placement of the neuraxial catheter took 45 minutes from OR entry to the completion of the procedure.

A 6-inch (15.2 cm), 17-G Tuohy epidural needle was used, and saline loss-of-resistance followed by cerebrospinal fluid (CSF) flow occurred at 13-cm depth at the L3/4 interspace. The 20-G catheter was advanced 6 cm into the intrathecal space. The catheter was then taped with the patient still in the sitting position. Eight people then repositioned the patient into a 45° semi-upright position. Oxygen supplementation of 3 L/minute via nasal cannula was begun, and the intrathecal catheter was dosed. Hyperbaric bupivacaine 12 mg, fentanyl 15 mcg, and morphine 100 mcg were given and saline was flushed in 0.25-mL increments over 12 minutes to gradually produce a T4 sensory blockade. During this interval, the patient was gradually lowered down to a 30° semi-upright position. Arterial pressure monitoring showed a lack of significant aortocaval compression despite no left uterine displacement. Motor block and sensory block height were assessed prior to each incremental dose. This 30° semi-upright position was then maintained throughout the procedure to minimize respiratory difficulty. Continuous positive airway pressure (CPAP) was immediately available intraoperatively. She breathed comfortably throughout the delivery, including with pannus tape retraction, maintaining SpO_2_ >95%. A supraumbilical, vertical skin incision followed by classical (vertical) hysterotomy was performed, as the obstetricians had determined that this approach would enable the best access to the uterus for safe delivery of her fetus. No additional local anesthetic was required, and the intrathecal catheter was removed without incident at the end of the procedure. Incision to closure time was 68 minutes, and the total time spent in the OR was 176 minutes. Postpartum, she recovered in the surgical intensive care unit and required CPAP while asleep. After 24 hours, she was moved to an inpatient ward with continuous ECG and SpO_2_ monitoring. Her postpartum course was uncomplicated, and she was discharged home on postpartum day four. She did not develop any postdural puncture headache (PDPH).

Second delivery

Eighteen months after her first delivery, our patient returned at 36 weeks and four days of gestation on the day of surgery for elective repeat cesarean (preterm due to her prior classical uterine incision) and bilateral tubal ligation. Her weight was 36 kg higher compared to her previous presentation, resulting in a BMI increase to 116. Her other medical history was unchanged. During her second pregnancy, her hematologist had stopped enoxaparin three days before cesarean due to a lack of thromboembolic events. Her higher BMI prompted additional considerations regarding delivery. The case was shifted to a non-obstetric OR to use an operating table that would support her weight (above the 300-kg obstetric table limit), and for the wider OR doorway to accommodate the inpatient recovery bed. A similar anesthetic approach was planned given that the slow-dose intrathecal catheter had been successful for the first delivery. To reduce time in the OR, her intrathecal catheter was placed prior to OR entry. Similar to the first delivery, she received ultrasound-guided placement of an arterial line and two 18-G peripheral IV lines.

The placement of the intrathecal catheter required 35 minutes, and the same sitting position and tape-facilitated landmark-based technique were successful. A 7-inch (17.8 cm), 17-G Gertie Marx epidural needle obtained CSF return at 15-cm depth, and a 19-G wire-wound, multi-orifice, flexible catheter was advanced 6 cm into the intrathecal space. In the OR, transfer to the table in a 45° semi-upright posture required the assistance of eight people. Supplemental oxygen of 4 L/minute via a simple face mask was started as the catheter was dosed. Again, hyperbaric bupivacaine 12 mg, fentanyl 15 mcg, and morphine 100 mcg were given in 0.25-mL increments over 15 minutes to gradually induce a T4 sensory blockade, and the patient was lowered to a 30° semi-upright position during this time. The 30° semi-upright position was maintained throughout the procedure. She had no respiratory difficulties during surgery. The surgical approach was similar to the first delivery: a supraumbilical midline skin incision and a classical uterine incision; the surgery proceeded uneventfully. The total surgical time was 92 minutes, and the total time spent in the OR was 131 minutes. During the closure, a preemptive dose of hyperbaric bupivacaine 1.875 mg (0.25 mL) was given prior to the removal of the catheter and the end of the case. She was transferred to a ward with continuous ECG and SPO_2_ monitoring, and she again required CPAP while asleep. Her recovery was again uncomplicated, and she was discharged home on postpartum day four never having had PDPH. The delivery details of the patient are summarized in Table 1.

**Table 1:** Case details during first and second cesarean deliveries LOR: loss of resistance; OR: operating room; CPAP: continuous positive airway pressure; ICU: intensive care unit

Variables	First delivery	Second delivery
Body mass index (kg/m^2^)	102	116
Weight (kg)	279	315
Height (cm)	165	165
Gestational age (weeks, days)	39, 2	36, 4
Urgency and indication for cesarean	Elective, breech presentation	Elective, previous classical hysterotomy
Anesthetic technique	L3/4 intrathecal catheter. Tape retraction, landmark-guided. Used 15-cm, 17-G Tuohy epidural needle. LOR 13 cm, threaded 6 cm	L3/4 intrathecal catheter. Tape retraction, landmark-guided. Used 17-cm, 17G-Gertie Marx^TM^ epidural needle. LOR 15 cm, threaded 6 cm
Neuraxial procedure (minutes)	45, in OR	35, out of OR
Anesthetic dose	Intrathecal: bupivacaine 12 mg, fentanyl 15 mcg, morphine 100 mcg; given in 0.25-ml increments, 0.25-ml saline flush, dosed over 12 minutes. To T4 level	Intrathecal: bupivacaine 12 mg, fentanyl 15 mcg, morphine 100 mcg; given in 0.25-ml increments, 0.25-ml saline flush, dosed over 12 minutes. To T4 level. Before closure: bupivacaine 1.875 mg (0.25 ml)
Surgical details	Semi-recumbent position, midline supraumbilical skin incision, classical (fundal) hysterotomy	Semi-recumbent position, midline supraumbilical skin incision, classical (fundal) hysterotomy
OR location	Obstetric unit	Main OR suite
Time from incision to closure (minutes)	68	92
Total time in OR (minutes)	176	131
Vasopressor intraoperative support	Phenylephrine infusion of 20-50 mcg/minute	Phenylephrine infusion of 25-50 mcg/minute
Estimated blood loss (ml)	700	800
Apgar score (1, 5 minutes)	1, 8	6, 9
Respiratory support	Intraoperative: nasal cannula, CPAP available. Postpartum: CPAP when asleep, continuous SpO_2_	Intraoperative: simple face mask, CPAP available. Postpartum: CPAP when asleep, continuous SpO_2_
Recovery unit	ICU for 24 hours then continuous SpO_2_-capable ward	Continuous SpO_2_-capable ward
Postdural puncture headache	No	No

## Discussion

This patient with a BMI >100 presented several challenges beyond those usually expected in parturients with morbid obesity closer to a BMI of 40 (Table 2). She benefitted from a predelivery consultation with an obstetric anesthesiologist [4,5]. This early consultation enabled coordination of a non-standard operative plan requiring the preparation of additional equipment, personnel, and extra scheduled time for her case. Predelivery consultation was also required to optimize her comorbidities and discuss perioperative management. The comorbidities she had are common in high-BMI individuals: OSA, asthma, pulmonary embolism, and hypertension [5,12]. Particular attention was paid to her anticoagulant plan and her pulmonary function. Her habitus caused respiratory difficulty in the usual surgical position, so it was important to discuss surgical positioning and the adjusted operative approach with the surgeon prior to bringing her to the OR. Restrictive ventilation is expected with very severe obesity and is further exacerbated by supine positioning [13]. Her semi-upright positioning was surgically feasible using a supraumbilical midline skin incision and classical uterine incision, as described in a previous case series [10].

**Table 2:** Anesthetic considerations for cesarean deliveries in morbidly obese parturients BMI: body mass index (in kg/m^2^); BP: blood pressure; IV: intravenous; CPAP: continuous positive airway pressure; OR: operating room; CSE: combined spinal-epidural; PDPH: postdural puncture headache; SpO_2_: pulse oximetry

Typical considerations with morbid obesity (BMI >40)	Additional considerations with BMI significantly higher than 50
Comorbidities require preoperative optimization and perioperative management	Pulmonary function and limitations verified in operative position(s)
Predelivery anesthesia consult for discussion of BMI-adjusted plan and risks	Semi-recumbent (30-45°) intraoperative position: coordinate with the surgeon, most ramps not high enough
Intrapartum cesarean more likely	Intraoperative CPAP discussed preoperatively and made available
Neuraxial placement likely to be more prolonged and/or difficult (or impossible), ultrasound may aid success	Longer time in OR (neuraxial block + positioning + surgical time) scheduled, anticipated in the anesthesia plan
Epidural block more likely to fail	OR table and perioperative bed(s) weight and dimension limits verified
Invasive BP monitor is common, and IV lines (and arterial line) often require ultrasound	Extra staff assigned for transferring and positioning
Ramp used for intraoperative position	Back-up anesthesia staff considered, in case of prolonged case
Anticipate, prepare for difficult mask or intubation	Longer neuraxial needles (epidural and spinal) made available
IV doses adjusted	Gradual, incremental dosing of the neuraxial catheter to minimize the risk of high block or respiratory distress
Difficult airway cart on hand	If sequential CSE is performed, consider lower spinal dose followed by gradual dosing of the catheter
	PDPH likely to be less common
	Postpartum care on unit with advanced capabilities: (invasive BP, continuous SpO_2_, CPAP)
	Multimodal analgesia planned, with escalation made available instead of relying on IV opioids

Prior to coming to the OR, the delivery team verified the operating table’s maximum patient weight and width. Patient transfers and positioning during the neuraxial catheter placement and the surgical procedure were exceptionally difficult and required a far larger number of assistants than is typical for less heavy patients. Spinal medication dosing was intentionally delayed until after the positioning was complete so that the patient could assist with movement as much as possible.

Lateral position for neuraxial block placement was considered given that this would minimize the shelf pannus where the back meets buttocks compared to the sitting position. Ultimately, the sitting position was chosen for block placement given the improved lateral distraction of tissue away from the midline, better visual estimation of the midline, and optimization of palpable landmarks. If spinous processes had not been palpable in this patient, we had planned to use ultrasound to identify her landmarks. Despite our success with palpation, evidence shows that ultrasound assistance reduces neuraxial procedure time and total needle passes in morbidly obese patients [14]. Notably, CSF return was observed at depths of 13 and 15 cm in this patient, which was greater than documented in a previous report of three patients with BMI ranges of 73-95 who had lumbar intrathecal catheter placement depths of 9-10 cm [10].

There is evidence from a randomized trial that the ED50 and ED95 of intrathecal bupivacaine are similar for morbidly obese patients (BMI >40) compared to patients with normal BMI, but that study did not parse out higher BMI subgroups [15]. We opted for incremental dosing by catheter followed by low-volume flushes, similar to the approach mentioned in a previous series, although our doses were given in smaller increments [10]. Most epidural catheters have 0.2-0.3-ml dead space [16]. The rationale for slow, incremental dosing in our patient was twofold: to minimize the risk of the high neuraxial blockade and to reduce the risk of actual or perceived respiratory decompensation. A retrospective study has reported high spinal in 2.5% of patients with BMI >50 versus 0.5% with BMI <50, although the magnitude of risk that correlates to those with very high BMI remains unclear [17]. Additionally, the use of hyperbaric local anesthetic plus the semi-upright position during dosing reduced the risk of high spinal. As mentioned, this patient could not breathe comfortably in the supine position even prior to the pregnancy. Restricted ventilation is expected to be exacerbated by surgical retraction and supine positioning, which we feared would induce respiratory distress requiring CPAP or intubation [13]. With these factors in mind, we opted for a gradual spinal blockade while monitoring her breathing pattern and slowly lowering her into her flattest-tolerated position.

In most instances, we place neuraxial blocks for cesarean delivery in the OR. Because of the prolonged time required for block placement and the time spent transferring and positioning during the patient’s first delivery, we chose to place the neuraxial catheter prior to coming to the OR for the second delivery in order to avoid wasted OR time. Ultimately, this strategy was effective, as the total OR time was shorter for the second surgery (131 versus 176 minutes) even though the total operative time was longer (92 versus 68 minutes) (Table 1). Having an obstetric OR occupied for an extended duration reduces its availability for other possible emergencies. This extended non-availability also applies to OR staff, especially during after-hours. These constraints may be less relevant at some hospitals.

Intrathecal catheters have many advantages as an anesthetic technique for very high-BMI parturients. They are highly reliable and use less local anesthetic than epidural catheters [reducing the risk of local anesthetic systemic toxicity (LAST) or the risk of high spinal in case of potential replacement] [18]. Although PDPH is a potential risk, studies suggest that PDPH may be reduced among those with BMI >30 [18,19]. Incidence of PDPH in patients with BMI very much higher than 50 remains unstudied, although no PDPH has occurred in any of the cases of cesarean patients with BMI >70 as reported in the literature [8-11]. The mechanism of lowered risk is uncertain, but theories proposed include increased abdominal pressure or epidural lipomatosis possibly compressing the intrathecal space and reducing leakage [19,20].

## Conclusions

This report adds to the sparse body of literature on anesthesia for cesarean delivery in very high-BMI individuals, and it is the first to detail neuraxial anesthetics in two consecutive cesarean deliveries for one woman with a BMI >100. We highlight the value of out-of-OR placement of an intrathecal catheter as well as the slow, incremental dosing of the catheter while adjusting the patient to a nonstandard, more upright surgical position for optimal respiratory effect. This case also highlights the additional considerations that are unique for very high-BMI cases: verification of operating table and gurney limits, tasking more staff than are usually required in the OR for transfer and positioning, a more upright surgical position, significantly longer times for all perioperative phases, and the importance of postpartum respiratory monitoring. The details of this rare case may help guide those who care for similar patients with regard to the preparation and management of the procedure.
